# Strategies to reduce morbidity following pleurectomy and decortication for malignant pleural mesothelioma

**DOI:** 10.1111/1759-7714.15067

**Published:** 2023-08-13

**Authors:** Patrick Bou‐Samra, Austin Chang, Kevin Zhang, Feredun Azari, Gregory Kennedy, Emily Guo, Wei‐Ting Hwang, Sunil Singhal

**Affiliations:** ^1^ Department of Thoracic Surgery University of Pennsylvania Perelman School of Medicine Philadelphia Pennsylvania USA; ^2^ University of Pennsylvania Perelman School of Medicine Philadelphia Pennsylvania USA; ^3^ Department of Biostatistics, Epidemiology, and Informatics (DBEI) University of Pennsylvania Philadelphia Pennsylvania USA

**Keywords:** morbidity, pleural mesothelioma, pleurectomy and decortication

## Abstract

**Background:**

Pleurectomy and decortication (PD) in malignant pleural mesothelioma has a high morbidity mostly associated with aspiration pneumonia (PNA), deep vein thrombosis (DVT), and foreign catheter sepsis. We instituted four strategies to reduce these complications and report our experience.

**Methods:**

This was a retrospective review of patients who underwent PD at the University of Pennsylvania between 2015 and 2022. Our patients underwent standard of care PD in addition to tracheostomy and gastrostomy/jejunostomy tube with therapeutic anticoagulation (AC) leading up to surgery. Measured outcomes were postoperative PNA, DVT, and sepsis. The predicted risk of those same outcomes had patients not undergone the interventions was calculated based on the American College of Surgeons (ACS) surgical risk calculator (SRC). A McNemar's test was used to determine whether the risk of having PNA, DVT and sepsis differed between the two subgroups.

**Results:**

Fifty‐five patients were included in the study. The mean age was 70 years (SD 6.2) with a mean of 21 (SD 19) pack‐years of smoking. PNA, DVT, and catheter‐related sepsis occurred in 12, four, and seven patients, respectively. Upon using the ACS SRC prediction model of the nonintervention group, PNA, DVT and catheter related sepsis was predicted to occur in 24 (paired data OR 5, 95% CI: 1.4–17.2; McNemar's test *p* = 0.008), 14 (paired data OR 3.5, 95% CI: 1.15–10.6; McNemar's test *p* = 0.03), and 17 (paired OR 3, 95% CI: 1.09–8.3; McNemar's test *p* = 0.04) patients, respectively.

**Discussion:**

Patients undergoing tracheostomy creation, therapeutic AC at the time of diagnosis, and gastrostomy tube placement had a reduced risk of aspiration PNA, DVT, and catheter sepsis.

## Graphical abstract

Pleurectomy and decortication for malignant pleural mesothelioma is associated with high post‐operative mortality associated with aspiration pneumonia (PNA), deep venous thrombosis (DVT) and line sepsis (associated with nutrition access). We observed that the creation of a tracheostomy, giving therapeutic anticoagulation at the time of diagnosis, and placement of a gastrostomy and jejunostomy tube were associated with a reduced the risk of aspiration PNA, DVT, and line sepsis in these patients.

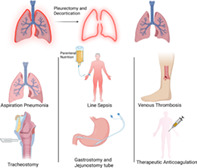

## INTRODUCTION

Malignant pleural mesothelioma (MPM) is an aggressive tumor that affects the lining of the lung and has an incidence of 1 in 100 000 persons in the United States.^1^ The current mainstay of management is resection of all grossly visible disease to achieve complete macroscopic resection.^2^

Pleurectomy and decortication (PD) is the removal of the parietal pleura accompanied by decortication of lesions on the lung pleura. PD is associated with several complications such as empyema, prolonged air leaks, and bronchial fistula. Postoperatively, the most common complications reported in the literature are pneumonia (PNA) in up to 21.4% of patients, deep venous thrombosis (DVT) in up to 28.6% of patients, and sepsis in up to 5.9%.^3^

Over the course of 8 years, we found that the most common postoperative causes of death in our cohort, similarly to our literature review, were PNA, DVT and catheter sepsis (central intravenous catheter and foley catheter). The catheter sepsis was usually associated with central access particularly for parenteral nutrition if the patient was in the ICU for a prolonged period of time. To actively reduce the risk of developing these complications, our group decided to institute four major changes in the surgical pathway: creation of a tracheostomy, insertion of a gastrostomy tube 2 weeks prior to surgery, insertion of a jejunostomy tube 2 weeks prior to surgery, and initiation of therapeutic anticoagulation (AC) during the preoperative period leading up to surgery.^4^ Our goal was to assess whether these four interventions were associated with a decrease in the prevalence of PNA, DVT and central line sepsis.

## METHODS

### Patient selection

Patients included in our study are those who underwent PD at the Hospital of the University of Pennsylvania between 2015 and 2022. These patients had met standard preoperative risk‐assessment metrics. Baseline characteristics and risk factors needed for risk calculation and clinical significance were collected including age, body mass index (BMI), smoking, renal disease, chronic obstructive pulmonary disease, lung cancer, asbestos exposure, Eastern Cooperative Oncology Group (ECOG) score, forced expiratory volume in 1 min (FEV1), forced vital capacity (FVC), and the receipt of neoadjuvant (NAT) and adjuvant therapy.

### Intervention

#### Intraoperative mini‐tracheostomy

At the conclusion of the operation, a 6 Fr mini‐tracheostomy was placed in the second tracheal ring. Our goal was to allow frequent bronchoscopies at the bedside, inflate the cuff to prevent aspiration of secretions, and reduce the need for any sedation should a patient need some transient ventilator support during recovery.

#### Therapeutic low molecular weight heparin

It has been shown that cancer patients are in a hypercoagulable state and could benefit from therapeutic AC in order to decrease the risk of DVT.^4^ As such, our patients were started on therapeutic low molecular weight heparin (LMWH) during the preoperative workup leading up to surgery in addition to subcutaneous heparin (SQH) postoperatively.

#### Preoperative gastrostomy tube and jejunostomy tube

All patients underwent a diagnostic laparoscopy 2 weeks prior to surgery with three major goals. The first goal was to identify any possible sources of metastatic disease to the abdomen which would preclude a PD. Second, a gastrostomy tube was placed to decompress the stomach and prevent aspiration of gastric contents. Third, a jejunostomy tube was placed. This allowed for rapid enteral nutrition the day following the PD surgery.

### Research design

#### The ACS NSQP risk calculator

The American College of Surgeons National Surgical Quality Improvement Program (ACS NSQIP) surgical risk calculator (SRC) is a collaborative effort from the American College of Surgeons that collects patient data and postoperative complications from more than 500 hospitals in the U.S. It assigns a risk‐adjusted 30‐day outcome and use it to guide clinical decision‐making and patient counseling.^5^ Preoperative risk factors are entered into the calculator with a specific current procedural terminology (CPT) code. The calculator then predicts individualized risks per complication and compares it to the average risk.

The c‐statistics were used to assess the predictive capacity of the ACS SRC in a study that involved 2514 patients undergoing pulmonary resections. A higher c‐statistic is associated with a better discriminatory capacity of the test and implies that patient experiencing the outcome were predicted to have a higher risk than those who did not. The c‐statistic for the development of any complication was 0.728, VTE was 0.792, and PNA was 0.715 indicating good predictive capacity.^6^, ^7^

To change our continuous calculated risk to a binary outcome, we focused on the concept that calculated risks below the average predicted risk per outcome were considered less likely to occur whereas those above the average were considered more likely to occur. In our study, we focused on the risk of development of PNA, DVT and sepsis. The CPT code we used was 32 320 which codes for decortication and parietal pleurectomy that all our patients underwent.

#### Matched group creation

All included patients had received all three interventions. Hence, the collected patient data and outcomes constitute our intervention subgroup. Our plan was to compare this cohort to a matched cohort whereby these interventions had not been implemented.

To establish a control group to determine if we had an improvement in outcomes, we considered three methods. First, we reviewed the literature to identify possible historical controls. However, due to statistical limitations and inconsistency in outcomes measured with our own measured outcomes, it was not possible to rigorously compare previous studies to our data. Second, we examined our own internal historical data. However, we felt this was too biased towards a single institution experience. Third, we ultimately opted for a rigorous and more patient‐specific approach, described below:

1. We utilized the SRC to calculate the risk of development of each outcome. This calculated risk is the individualized patient risk after PD had there been no intervention.
2. Our collected patient data had a binary outcome for PNA, DVT, and sepsis. Meanwhile, the data representing the matched pair group was a predicted risk. A cutoff was needed to change that risk into a binary outcome. A risk higher than the cutoff is likely a situation where the complication occurred and a risk lower than the cutoff is likely a situation where the complication had not occurred.
3. Each procedure has an average predicted risk per complication by the SRC, which has been shown to have a good predictive capacity.^6^, ^7^ We used that risk as our cutoff (2.1%, 2.2%, 1.5% for PNA, DVT and sepsis, respectively). Patients having a risk higher than that were considered to have developed the outcome of interest while those with a lower risk did not.

### Statistical analysis

Descriptive statistics are provided for baseline patient characteristics. The outcomes of interest in this study are the binary outcomes of the development of PNA, DVT, or sepsis following the interventions previously mentioned. First, frequency and proportion of outcomes were computed. Then, to compare the observed outcome to the predicted outcomes based on the calculated SRC risk scores, McNemar's test was used. Due to the small sample size, the exact McNemar's test was used. Then, among those patients with a change in outcome, an odds ratio was calculated to understand in what direction the intervention studied changed the outcome.

Odds ratios for the match‐paired data and the 95% CI were calculated. A *p*‐value of <0.05 was considered statistically significant. SPSS version 28 (IBM Statistics) was used for our statistical analysis.

## RESULTS

### Patient characteristics

The mean age of our population was 70 years (SD: 6; range: 55–83), with a mean BMI of 27.7 kg/m^2^ (SD: 3.9). On average, they had 21 pack‐years of smoking (SD 19). The mean FEV1 was 0.75 L (median 0.72) and mean FVC was 0.75 L (median 0.7). There were 43 males and 12 females, most patients were ASA 3 (53/55), 23.6% had diabetes, and 76.4% had a history of asbestos exposure. Most patients had an ECOG score of 1; 36/55 (65.5%), 14.8% underwent NAT and 40% underwent adjuvant therapy (Table 1).

**TABLE 1:** Baseline characteristics of cohort.

Patient Characteristics		*N* = 55
Age (mean‐SD)		70.2 (6.2)
BMI (mean‐SD)		27.70 (3.9)
Sex	M	43 (78.2%)
	F	12 (21.8%)
Ascites within 30 days	No	54 (98.2%)
	Yes	1 (1.8%)
Steroid use	No	52 (94.5%)
	Yes	3 (5.5%)
ASA class	1	0
	2	0
	3	53 (96.4%)
	4	2 (36.4%)
Diabetes	No	42 (76.4%)
	Yes	13 (23.6%)
HTN requiring medication	No	22 (40%)
	Yes	33 (60%)
Dyspnea	No	34 (63%)
	Yes	20 (37%)
Family history of lung cancer	No	44 (80%)
	Yes	11 (20%)
Smoking pack‐years (mean‐SD)		21.12 (19)
Asbestos exposure	No	13 (23.6%)
	Yes	42 (76.4%)
ECOG	0	18 (32.7%)
	1	36 (65.5%)
	2	1 (1.8%)
	3	0
FEV1 (L, mean‐SD)		0.75 (0.25)
FVC (L, mean‐SD)		0.75 (0.33)
Adjuvant therapy	No	14 (25.9%)
	Yes	40 (74.1%)
Neoadjuvant therapy	No	46 (85.2%)
	Yes	8 (14.8%)

Abbreviations: ASA, American Societ of Anethesiology; BMI, body mass index; ECOG, Eastern Cooperative Oncology Group; FEV1, forced expiratory volume in 1 minute; FVC, forced vital capacity; HTN, Hypertension; L, liters.

### Predicted risk of PNA, DVT and sepsis

The predicted risks based on the SRC were those where the patients would not have received the previously mentioned interventions. For PNA, the mean predicted risk was 2.0% (SD 0.8; range: 0.9–4.6). The mean predicted risk of DVT development was 2.12% (SD 0.36; range: 1.4–3.2). The mean predicted risk of sepsis was 1.67% (SD 0.5; range: 1–3.4).

Using the average risk of developing the complication in question based on the SRC, we converted the continuous risk to a binary outcome and calculated the prevalence of each complication in the predicted group and compared it to those which were actually observed (Table 2).

**TABLE 2:** Observed and predicted outcomes based on the cutoff values.

Outcomes	Patients undergoing intervention	Predicted matched pairs had they not undergone interventions
PNA	12/55 (21.8%)	24/55 (43.6%)
DVT	4/55 (7.3%)	14/55 (25.5%)
Sepsis	7/55 (12.7%)	17/55 (30.9%)

Abbreviations: DVT, deep vein thrombosis; PNA, pneumonia.

### Tracheostomies are associated with decreased prevalence of PNA among patients undergoing PD

First, patients who had received a tracheostomy were weaned off the ventilator within 4 h. Furthermore, McNemar's test showed that there was a statistically significant difference in the observed incidence of PNA and the one predicted by the ACS SRC (which assumes no tracheostomy was performed) (McNemar's test, *p* = 0.008). There was a decrease in the total number of patients developing PNA following tracheostomy from 24 in the control group to 12 in the intervention group (Table 2).

Following tracheostomy creation there was a change in the outcome of 18 patients. 15/18 patients (83.3%) without tracheostomy creation were predicted to have PNA but did not upon tracheostomy creation. Meanwhile, 3/18 (16.7%) that were predicted to not develop PNA, did develop it upon tracheostomy creation. The likelihood of developing PNA without tracheostomy creation was estimated to be five times of the likelihood of a patient who underwent tracheostomy creation (matched‐pair OR: 5.0, 95% CI: 1.4–17.2) (Table 3).

**TABLE 3:** Comparison of the predicted risk of pneumonia (PNA) without a tracheostomy with the observed PNA incidence following tracheostomy creation.

Observed event with tracheostomy	Developed PNA	Did not develop PNA	Total
Predicted event without tracheostomy			
Developed PNA	9	15	24
Did not develop PNA	3	28	31
Total	12	31	55

### Therapeutic DVT prophylaxis is associated with a decreased incidence of VTE among patients undergoing PD

A McNemar's test showed that there was a statistically significant difference in the observed incidence of DVT and the one predicted by the ACS SRC (which assumes no therapeutic LMWH was given) (McNemar's test, *p* = 0.03). There was a decrease in the total number of patients developing DVT following therapeutic LMWH from 14 in the control group to four in the intervention group (Table 2).

Following LMWH administration, there was a change in the outcome of 18 patients. A total of 14/18 (77.8%) patients without therapeutic LMWH were predicted to have DVT but did not upon therapeutic LMWH administration. Meanwhile, only 4/18 patients (22.2%) that were predicted to not develop DVT did develop it upon therapeutic LMWH administration. The likelihood of developing DVT without therapeutic LMWH was estimated to be 3.5 times of the likelihood of a patient who received therapeutic LMWH (matched‐pair OR: 3.5, 95% CI: 1.15–10.6) (Table 4).

**TABLE 4:** Comparison of the predicted risk of deep vein thrombosis (DVT) without therapeutic low molecular weight heparin (LMWH) with the observed DVT incidence following therapeutic LMWH.

DVT prophylaxis	Developed DVT	Did not develop DVT	Total
No DVT prophylaxis			
Developed DVT	0	14	14
Did not develop DVT	4	37	41
Total	4	51	55

### Gastrostomy tube placement is associated with a decreased incidence of line sepsis among patients undergoing PD

A McNemar's test showed that there was a statistically significant difference in the observed incidence of sepsis and the one predicted by the ACS SRC (which assumes no gastrostomy tube was placed for nutrition) (McNemar's test, *p* = 0.04). There was a decrease in the total number of patients developing line sepsis following therapeutic gastrostomy tube placement from 17 in the control group to seven in the intervention group (Table 2).

Following gastrostomy tube placement, there was a change in outcome of 20 patients. Fifteen out of 20 patients (75%) without gastrostomy tube placement were predicted to have sepsis but did not upon its placement. Meanwhile, only five out of 20 (25%) that were predicted to not develop sepsis, did develop it. The likelihood of developing sepsis without gastrostomy tube placement was estimated to be three times the likelihood of a patient who had one placed (matched‐pair OR: 3, 95% CI: 1.09–8.3) (Table 5).

**TABLE 5:** Comparison of the predicted risk of sepsis without gastrostomy placement with the observed sepsis incidence following gastrostomy placement.

Percutaneous gastrostomy Tube (PEG) creation	Developed sepsis	Did not develop sepsis	Total
No PEG creation			
Developed sepsis	2	15	17
Did not develop sepsis	5	33	38
Total	7	48	55

Abbreviation: PEG, Percutaneous gastrostomy Tube.

## DISCUSSION

Mesothelioma is a devastating disease with virtually no cures. Surgery aims to debulk disease while allowing a meaningful recovery. Unfortunately, the operation has been plagued by a high rate of morbidity and mortality. The purpose of this study was to identify strategies that may reduce surgical complications that has major consequences including increased pain, suffering, death and reduced likelihood of obtaining postoperative chemotherapy and/or radiation which are also important parts of the total treatment strategy.

To address these complications, we evaluated the literature, our own historical data, and our current patient outcomes. We noticed a significantly high mortality secondary to PNA, DVT, and line sepsis. The PNA was typically due to aspiration given the bronchoalveolar lavage culture that showed a predomination of anaerobic organisms. This data resembles the reported literature. Study have shown that prevalence of PNA following lung resection for noninfectious disease was around 25%. It is associated with a 19%–40% mortality in the thoracic subgroup, longer hospitalization and need for an ICU admission.^8^, ^9^ On a different note, up to 40% of patients undergoing major general surgery in Western populations develop a DVT.^10^ To solve this problem, we considered several strategies.

First, we realized that one of the major factors that causes aspiration pneumonia was the loss of the phrenic nerve and diaphragmatic dysfunction which is common after PD.^11^ Loss of the phrenic nerve leads to reduced cough and secretion clearance. Hence, patients may not be able to recover from micro‐aspiration events that likely occur in the immediate postoperative period due to altered sensorium. Furthermore, if a patient does need ventilator support, there is a reluctance to go back on a ventilator which often tires the patient. It can become an urgent event which includes rapid infusion of paralytics and sedatives which further worsens the situation. We postulated that a mini‐tracheostomy placed at the time of surgery may overcome this issue.

It also allows for bronchoscopy for secretion management the first several days while the patient is recovering and avoids sedatives. We simply instill 10 mL of lidocaine through the tracheostomy a minute prior to the bronchoscopy. If a patient does need ventilator support, we can give continuous positive airway pressures (CPAP) through the tracheostomy without sedatives or paralytics. Also, the ICU staff are more comfortable when they know airway access is not an issue.

Second, loss of phrenic nerve can also lead to stomach distention and elevation of the location of the lower esophageal sphincter. In the postoperative period when the patient is often sedated or neurologically impaired, the risk of aspiration is high even with precautions such as head of bed elevation and a nasogastric tube.^12^ Therefore, we felt a gastrostomy tube would be beneficial in fixing the stomach, reducing gastric contents and avoiding a nasogastric tube.

Third, while the gastrostomy tube addresses aspiration, malnourishment remains a serious concern in cancer patients. It has been reported that patients who were malnourished had a reduced ability to complete cancer treatment.^13^ There is a plethora of data that early feeding and the restoration of gut microbe improves wound healing, decreased postoperative length of stay, lower overall complication rate and even lower mortality rate.^14^ As few as 12% of patients in some case series undergoing lung cancer surgery are able to commence enteral nutrition postoperatively.^15^ We hypothesized that a jejunostomy tube at the time of the gastrostomy tube would allow rapid enteral access. Also, the presence of a jejunostomy tube allows instilling medications and avoiding the need for a central vascular access, which has been shown to be an independent risk factor for central line‐associated bloodstream infections.^16^ Another advantage is that we frequently do perform a laparoscopy prior to surgery to place the jejunostomy tube which simultaneously allows for evaluation of silent peritoneal mesothelioma in patients with pleural mesothelioma.^17^

Fourth, we noticed that a DVT and subsequent pulmonary emboli is devastating to a postoperative PD patient. It is likely that patients who have bulky mesothelioma have two factors: increased intrathoracic pressure and a higher‐than‐average risk of Virchow's triad leading to DVTs. We believe that the increased intrathoracic disease from mesothelioma leads to a small change in venous return, which, in combination with Virchow's triad, leads to venous stasis. Many of these small DVTs may not be immediately discovered by lower extremity dopplers (e.g., iliac vein thrombosis). Therefore, we opted to start all patients who were being considered for PD with preoperative therapeutic LMWH. We discontinued this 3 days before surgery so we could place an epidural for surgery. After surgery, we resumed standard‐of‐care low dose prophylactic anticoagulation. Interestingly, there is evidence that heparins may inhibit tumor cell growth, invasion, and distant metastasis but the studies at this time are not powered enough to make generalizable conclusions.^18^ In fact, a recent American Society of Clinical Oncology (ASCO) update recommended that patients with cancer undergoing major surgery should receive prophylaxis starting before surgery and continuing for at least 7–10 days.^19^

This study had certain limitations. We were limited by sample size given the rarity of this tumor and the nature of this single‐center study. We were also limited by the fact that we were unable to control for our patients within the same hospital and at a time prior to the implementation of these interventions. However, we utilized the SRC to create a control group that resembled the intervention group the most. When it comes to the outcomes studied, we used tracheostomies to address aspiration PNAs. The SRC describes PNA in general and does not specify granular data on whether it is aspirational or not. Similarly, the sepsis we reported in our patients is related to line sepsis. The SRC reports the risk to develop sepsis in general and does not specify the setting in which it developed. Another important consideration is tumor stage. Our cohort was heterogenous in terms of stage (stage I–IIB). There is evidence that patients at a more advanced stage have a lower life expectancy.^20^ However, there is no clear evidence that surgical patients with more advanced stages would necessarily have more postoperative complications. As such, we did not include stage as a variable in our study. Furthermore, the current SRC does not include tumor stage in the required preoperative data but rather the presence or absence of malignancy.

In conclusion, we observed that creation of a tracheostomy, giving therapeutic anticoagulation at the time of diagnosis, and placement of a gastrostomy and jejunostomy tube were associated with a reduced the risk of aspiration PNA, DVT, and line sepsis in patients with MPM undergoing PD. Larger multi‐institutional studies are needed especially with diseases as rare as MPM to further enhance the perioperative care of these patients.

## AUTHOR CONTRIBUTIONS

Patrick Bou‐Samra: Conceptualization, data curation, formal analysis, investigation, methodology, software, writing original draft, writing review and editing. Austin Chang: Data curation, investigation, writing review and editing. Kevin Zhang: Data curation, investigation, writing review and editing. Feredun Azari: Conceptualization, investigation, methodology, writing review and editing). Gregory Kennedy: Conceptualization, investigation, methodology, writing review and editing). Emily Guo: Conceptualization, data curation, writing review and editing. Wei‐Ting Hwang: Conceptualization, formal analysis, investigation, methodology, supervision, writing review and editing. Sunil Singhal: Conceptualization, formal analysis, investigation, methodology, supervision, writing original draft, writing review and editing.

## CONFLICT OF INTEREST STATEMENT

There are no conflicts of interest or financial disclosures for any of the authors listed above. This study has not been submitted or is under consideration elsewhere.
